
JOURNAL INFORMATION
==============================
NLM Title Abbreviation: ScientificWorldJournal
Journal ID: ScientificWorldJournal
NLM Journal ID: 101131163
Journal ID: TSWJ

The Scientific World Journal

ISSN: 2356-6140
EISSN: 1537-744X
Publisher: Wiley

ARTICLE INFORMATION
==============================
PMCID: PMC5848710
PMID: 19030760
DOI: 10.1100/tsw.2008.150
Article ID: 812607
Article version: 1
Subjects: Clinical Image

Adult Intussusception Presenting with Target Sign

Gorospe Emmanuel C. *
Department of Medicine, Johns Hopkins Bayview Medical Center, Johns Hopkins University School of Medicine, Baltimore, MD, USA
*Emmanuel C. Gorospe: egorosp1@jhmi.eduAcademic Editor: Daniel Batlle

Publication date: 2008
Electronic publication date: 2008 Nov 22
Volume: 8
First page: 1154
Last page: 1155
Received 2008 Sep 20; Accepted 2008 Nov 14
Copyright: Copyright © 2008 Emmanuel C. Gorospe.
Copyright year: 2008
License: This is an open access article distributed under the Creative Commons Attribution License, which permits unrestricted use, distribution, and reproduction in any medium, provided the original work is properly cited.
License URL: https://creativecommons.org/licenses/by/3.0/

Keywords: intussusception, ileocolic, computed tomography

==============================
Clinical Image
*TheScientificWorldJOURNAL* (2008) 8, 1154–1155
ISSN 1537-744X; DOI 10.1100/tsw.2008.150

TheScientificWorldJOURNAL
www.thescientificworld.com

### Adult Intussusception Presenting with Target Sign

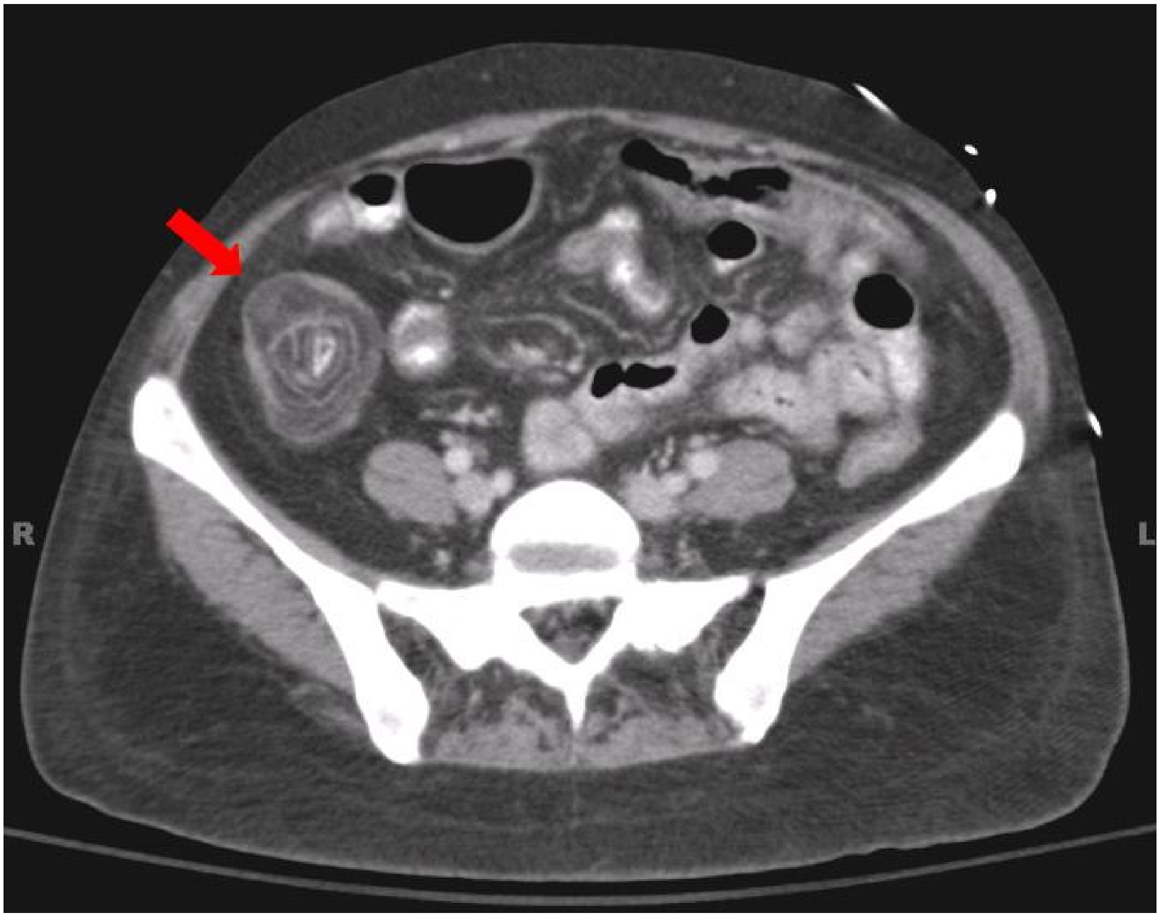

**FIGURE**. CT of the abdomen with target sign, suggesting ileocolic intussusception (see arrow).

Emmanuel C. Gorospe
*Department of Medicine, Johns Hopkins Bayview Medical Center, Johns Hopkins University School of Medicine, Baltimore, MD*

**E-mail**: egorosp1@jhmi.edu

*Received September 20, 2008; Accepted November 14, 2008; Published November 22, 2008*

**KEYWORDS**: intussusception, ileocolic, computed tomography

©2008 with author.
Published by TheScientificWorld; www.thescientificworld.com

Intussusception occurs when a proximal bowel segment telescopes into a contiguous distal portion. It is a rare condition in adults, occurring in less than 1% of all intestinal obstructions[1]. Spontaneous or idiopathic intussusception in adults is even rarer. Unlike in children, an underlying cause is present in 90% of cases due to intraluminal lesions, malignancy, adhesions, and postsurgical complications[2,3,4]. This abdominal CT is from a 48-year-old female who presented with a 2-day history of worsening right-sided abdominal pain, nausea, and loss of appetite. There was no previous abdominal surgery or other medical comorbidities. Her screening colonoscopy 4 months ago was unremarkable. The physical exam was significant for right-sided abdominal tenderness. The leukocyte count was 21,500/µL and lactic acid was 23 mg/dL. The abdominal CT scan showed a classic "target" or "donut" sign, suggestive of ileocolic intussusception. The patient underwent partial right colon resection with end-to-end anastomosis. The resected small bowel and colon segments showed ischemic changes only. The patient was discharged without complications. Abdominal CT is the imaging of choice[5]. When diagnosed, surgical resection is preferred compared to reduction in view of multiple possible pathology[3].

### REFERENCES

1. Triantopoulou, C., Vassilaki, A., Filippou, D., Velonakis, S., Dervenis, C., and Koulentianos, E. (2004) Adult ileocolic intussusception secondary to a submucosal cecal lipoma. *Abdom. Imaging* **29**, 426–428.
2. Weilbaecher, D., Bolin, J.A., Hearn, D., and Ogden, W., 2nd (1971) Intussusception in adults. Review of 160 cases. *Am. J. Surg*. **121,** 531–535.
3. Yalamarthi, S. and Smith, R.C. (2005) Adult intussusception: case reports and review of literature. *Postgrad. Med. J.* **81,** 174–177.
4. Jabar, M.F., Prasannan, S., and Gul, Y.A. (2005) Adult intussusception secondary to inflammatory polyps. *Asian J. Surg*. **28,** 58–61.
5. Takeuchi, K., Tsuzuki, Y., Ando, T., Sekihara, M., Hara, T., Kori, T., and Kuwano, H. (2003) The diagnosis and treatment of adult intussusception. *J. Clin. Gastroenterol*. **36,** 18–21.

**This article should be cited as follows:**

Gorospe, E.C. (2008) Adult intussusception presenting with target sign. *TheScientificWorldJOURNAL* **8**, 1154–1155. DOI 10.1100/tsw.2008.150.
